# Effects of a Therapeutic Horseback Riding Program on Social Interaction and Communication in Children with Autism

**DOI:** 10.3390/ijerph18052656

**Published:** 2021-03-06

**Authors:** Mengxian Zhao, Shihui Chen, Yonghao You, Yongtai Wang, Yanjie Zhang

**Affiliations:** 1 School of Physical Education, Shenzhen University, Shenzhen 518060, China; helenzhao2020@126.com; 2 Department of Kinesiology, Texas A&M University, Texarkana, TX 75503, USA; 3 Department of Sports Science, Hefei Normal University, Hefei 230061, China; hao2703@163.com; 4 College of Health Sciences and Technology, Rochester Institute of Technology, Rochester, NY 14623, USA; ytwchst@rit.edu; 5 School of Humanities and Social Science, Chinese University of Hong Kong, Shenzhen 518060, China

**Keywords:** autism spectrum disorder, animal-assisted intervention, therapeutic horseback riding, social interaction, communication skills

## Abstract

Various therapeutic interventions have been studied and found to be effective in reducing the stereotypical behaviors of children with autism spectrum disorder (ASD). There has been increasing interest in using animal-assisted interventions (AAIs) as an alternative approach to therapeutic rehabilitation for children with ASD, and many studies have reported that AAI has significant benefits for the cognitive, psychological, and social behavior of children with ASD. The present study was designed to examine the effects of a 16 weeks therapeutic horseback riding program on social interaction and communication skills in children with autism. Eighty-four children diagnosed with ASD, aged between 6 and 12 years old, were recruited for this study. All selected participants met the DSM-V criteria, and a total of sixty-one participants (*N* = 61) completed the study. A quasi-experimental design with an experimental group and control group was implemented for this study, taking measurements at pre-test, interim-test, and post-test to monitor the behavior changes in social and communication throughout the 16-week intervention. Repeated measures ANOVA and the independent sample *t*-test were used for data analysis, to assess the difference between the experimental group and control group. The results indicated that the THR program had positive influences on overall social skills and communication, based on the SSIS and the ABLLS-R scores, compared to the control group (*p* < 0.05). A notable improvement in the overall social interaction score was observed from the interim-testing point to post-test. In addition, participants in the therapeutic horseback riding (THR) group achieved significant improvements on six out of seven items in their communication evaluations. In conclusion, after 16 weeks of intervention, the THR program significantly enhanced the subdomains of social and communication skills in the areas of social interaction, communication, responsibility, and self-control, compared to the control group.

## 1. Introduction

Children with autism spectrum disorder (ASD) typically have impairments in social interaction and communication and show stereotyped behavior patterns. Today, the number of children diagnosed with ASD is increasing dramatically around the world. An estimated 1 in 54 children had ASD in the United States in 2020, which is nearly a 170% increase from the 1 in 150 with this diagnosis in 2000 [1,2]. There is also an increasing trend in the prevalence of children with ASD in Asia. Therefore, the educational approach and behavior therapies for children with ASD have attracted increasing attention over the past decade.

Many therapeutic interventions have been studied and found to be effective for certain typical behaviors of children with ASD, particularly in improving autistic children’s social interactions and communication skills [3,4]. Recently, there has been increasing interest in animal-assisted interventions (AAIs) as an effective option for therapy for children with ASD. Research studies have indicated that AAI can be used as a structured and goal-directed intervention which connects children with ASD to nature, the environment, and animals through structured activities [5]. Studies show that AAI may be an alternative treatment to traditional interventions for children with ASD.

Research studies have also found that AAI has significant benefits in the cognitive, psychological, and social domains for children with ASD [5,6]. Interactions with animals (i.e., dogs, horses, dolphins, rabbits, guinea pigs, llamas) can enhance psychosocial wellbeing [7], reduce stress, the heart rate, blood pressure, loneliness, and isolation, [8] and increase social interaction and socio-emotional functioning [9,10].

Therapeutic horseback riding (THR), which is one kind of animal-assisted intervention, is an alternative treatment which provides certain activities on horses to build a therapeutic interaction and communication between the riders and horses [11,12]. THR may have positive effects on multiple impairments of children with ASD, including physical, emotional, social, cognitive, behavioral, and educational functioning [11,12]. There is also evidence for it improving motor functioning and sensory processing in children with ASD [13,14,15,16]. Moreover, it has been reported that THR can positively affect children’s mental well-being, self-efficacy, and self-esteem, and thus significantly influence the quality of life of children with ASD [13,17,18,19].

As one of the core characteristics of ASD, deficits in social interaction and communication may lead to social isolation and withdrawal behavior, which can directly impact on personality and social development. Therefore, an AAI program focusing on social and communication skills is imperative to the development and quality life of children with ASD [20,21,22]. THR may provide a multisensory stimulating environment and therapeutic interactions through child and horse interplaying activities that are beneficial to children’s social and communication ability [23].

Today, the support for THR interventions for children with ASD is still limited by a number of methodological weaknesses in the research (e.g., lack of a control condition; small sample size; or single data source). Moreover, little evidence is available about use of a structured THR program as an intervention for the core behavior impairments of children with ASD. Currently, few quantitative studies with larger sample sizes, randomized control trials, and long-term interventions with THR have been conducted, especially in China. Thus, the THR program of this study created a structured routine in which horseback riding tasks were broken down into different steps, incorporating visual card supports, as well as utilizing verbal or nonverbal scripts in order to assist the participants with getting involved immediately and actively. It was inferred that a highly structured program could capture the children’s attention and generate a sustained level of focus. With this context, the purpose of this study was to examine the effects of a 16-week therapeutic horseback riding program on social interaction and communication skills in children with ASD. It was hypothesized that children with ASD in the THR intervention would demonstrate an improvement in social interaction and communication skills after the 16 week intervention, compared to participants in the control group who participated in the routine activities of a program for children with ASD.

## 2. Materials and Methods

### 2.1. Participants

**Figure 1:**
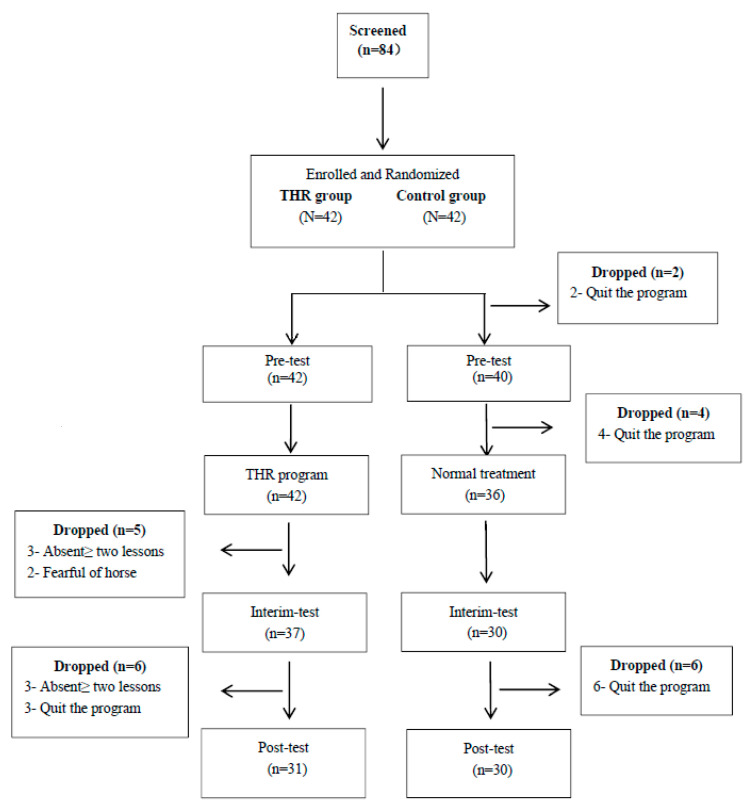
Participant flow diagram.

Eighty-four children diagnosed with ASD, aged from 6 to 12 years old, were recruited from therapy centers and special schools for children with ASD. All selected participants met the DSM-V criteria [24], and informed consent was obtained from each caregiver or parent before the intervention. Forty-two participants were randomly assigned to the experimental group, in which a therapeutic horseback riding program was implemented as the intervention group. The other forty-two children participated in regular activities as the control group. During the whole program, fifteen children quit the program for different reasons, and eight children had to withdraw the program because of absences and fear of horses. A total of sixty-one participants (*N* = 61) completed the THR program. An independent sample *t*-test was conducted to compare the baseline data between the experimental group and control group. There were no significant differences between the two groups in terms of the participants’ age, gender, and diagnoses. The demographic variables of participants are listed in Table 1 (*p* > 0.05). The flow diagram below (Figure 1) illustrates the selection process for the participants.

### 2.2. Procedures

The design of present study using a randomized experimental group and control group was to examine the effectiveness of a 16-week therapeutic horseback riding program on social and communication skills in children with autism. The study took measurements at three time intervals, the pre-test, interim-test, and post-test, to monitor participants’ behaviors in social and communication throughout the 16-week intervention. The pre-test was started one week before the experiment, the interim-test was conducted at the 8th week, and the post-test was conducted immediately after the 16-week.

### 2.3. Measurements

The Social Skills Improvement System Rating Scales (SSIS-RS), which includes five subscales (communication, cooperation, assertion, responsibility, empathy, engagement, self-control) was used to evaluate children with social and behavioral difficulties [25]. In the current study, the teachers of the training center in the program completed the SSIS-RS three times for the children’s social behavior assessments. The Assessment of Basic Language and Learning Skills-Revised (ABLLS-R) was used to evaluate social interactions from the perspectives of parents in this study [26].

### 2.4. Therapeutic Horseback Riding Program (THR Program)

The THR program took place at the International Equestrian Training Center in Jinan (Shandong Province, China). The center has two outdoor arenas and an indoor arena. The 16-week THR intervention program was conducted twice a week, with a total of 32 sessions lasting approximately 60 min. Each THR session was a structured program addressing individual therapeutic goals and development. The THR program involved a cooperative therapeutic team, which consists of horses, certified therapeutic riding instructors, and trained volunteers.

The THR sessions consisted of structured activities and exercises that addressed social skills, communication skills, and horsemanship skills. Each THR session followed the same routine during the 16-week intervention: (a) warm-up activities; (b) riding skills and horsemanship skills instruction; (c) THR exercises and activities; and (d) cool down and reward activities. The THR sessions were conducted in small groups. All the participants were led by riding instructors, with trained volunteers who worked with the same participant and the same horse throughout the whole 16-week program to promote horse-rider relationship-building. The horses were assigned according to the physical size and ability of the participants. Before the intervention, all the volunteers participated in training classes, and most of them played the role of “side walkers”. The riding instructors and side walkers closely monitored the children’s behavior to ensure the safety of the practice.

All learning processes were designed based on the aims of this study, and many purposely structured activities were incorporated to generate more opportunities to improve the targeted behaviors of participants. In each session, participants were advised to follow the instructors’ verbal and nonverbal instructions to interact with the horse. For example, during a session, each participant was “forced” to interact with the horse, respond to instructors (high-five to instructors, say bye to the horse), and naturally formed new interactions and communicated with others (physical, verbal, or eye contact). When the participants followed the instructions and accomplished the activities in each session, it was inferred that participants had practiced and finished a brief phase of socialization. Furthermore, the participants routinely performed the same series of activities and exercises to strengthen/improve their riding skills, to achieve their goals (goal-oriented). During the sessions, visual tools (pictures, cards, colorful drawings) were used in order to help the children understand clearly what they should do next. Table 2 shows the THR intervention program protocol.

### 2.5. Data Collection and Analysis

In the present study, a quasi-experimental design with repeated measures over three time intervals was utilized to evaluate the effects of the THR program on the social and communication skills of children with autism. Data collection was conducted by the instructors and parents, through administering the SSIS-RS and ABLLS-R at the three time intervals (pre-test, interim-test, and post-test). The data of the two instruments were analyzed through the repeated measures ANOVA to determine the differences in social interaction and communication skills between the experimental and control group after 16 weeks of therapeutic horse-riding intervention. All statistics analyses were conducted using the SPSS 25.0 software package (IBM Corporation, Chicago, IL, USA). Significance was two-tailed at *p* < 0.05.

## 3. Results

### 3.1. The Effects of the Therapeutic Horseback Riding Program on Social Interaction

#### 3.1.1. The Effects of the Therapeutic Horseback Riding Program on the SSIS

The Social Skills Improvement System Rating Scales was employed to evaluate the social skills of children with ASD, and a series of repeated ANOVAs was used on the final sample to assess possible changes in social interactions among children with ASD in the THR experimental group compared to the control group (Mauchly’s test of sphericity: Mauchly’s W = 0.450, *p* < 0.05) (see Table 3).

The results from the Table 3 show that there were significant differences in terms of time: F = 38.874, *p* < 0.05, and time x group interaction: F = 21.057, *p* < 0.05. The results of the multivariate tests showed that the time factor had a significant effects (*p* < 0.05) on social skills over time, and the interaction between testing intervals (interim-test and post-test) and groups (experimental and control groups) also showed a statistically significant difference (*p* < 0.05). Therefore, the results of the data analysis showed that the social skills of children with autism in the THR group were significantly improved after the 16-week intervention.

Repeated measures ANOVAs were conducted to assess whether the THR program could achieve an improvement in social skills between the two groups across the pre-test, interim-test, and post-test. As shown in Table 4, there was no significant difference between groups on the SSIS before intervention. In the interim test, there was a significant improvement for the experimental group compared with the control group (*p* < 0.05). After the 16 weeks, a significant improvement in social skills was continuing demonstrated for the THR program group compared with the control group (*p* < 0.01).

**Figure 2:**
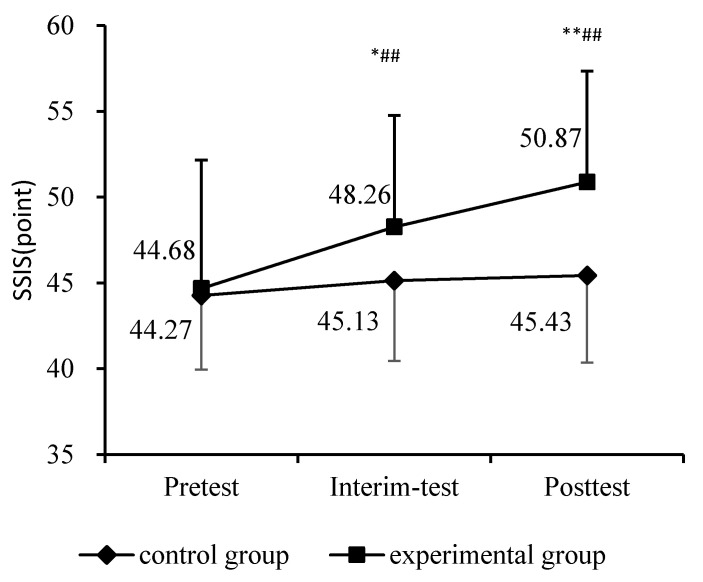
Results of THR program on the SSIS between two groups. Notes: Compared with the control group, * *p* < 0.05; ** *p* < 0.01. Compared with pre-test, ^##^ *p* < 0.01.

Participants in the THR group showed significantly improvements in their overall SSIS score over time from pre-test to post-test, and Figure 2 illustrates the trend of improvement in social skills for the two groups. The THR group had a more noticeable improvement on the SSIS compared to the control group, which indicated that the program had a positive influence on the social skills of children in THR group.

The study also evaluated whether the THR program demonstrated an improvement in the seven sub-areas of the SSIS between the experimental and control groups across three testing intervals. As shown in Table 5, greater improvements were observed in the items of communication, responsibility, and self-control for the THR group over time compared with control group (*p* < 0.01). These results showed that the THR program significantly improved the scores in the areas of communication, responsibility, and self-control sub-areas of social skills on the SSIS after the 16-week intervention.

#### 3.1.2. The Effects of the Therapeutic Horseback Riding Program on the ABLLS-R

The ABLLS-R was also used to examine the social skills of children with ASD, and a series of repeated ANOVAs was adopted on the final sample to assess possible changes in social interaction among children with ASD in the THR group, compared to children in the control group (Mauchly’s W = 0.884, *p* < 0.05). As reported in Table 6, the time effect was statistically significant, Pillai’s Trace = 0.684, F = 62.915, *p* < 0.05, along with the time x group interaction effect, Pillai’s Trace = 0.517, F = 31.076, *p* < 0.05. The data indicated that the social interaction scores of participants in the THR group were significantly improved through the 16-weeks intervention.

**Figure 3:**
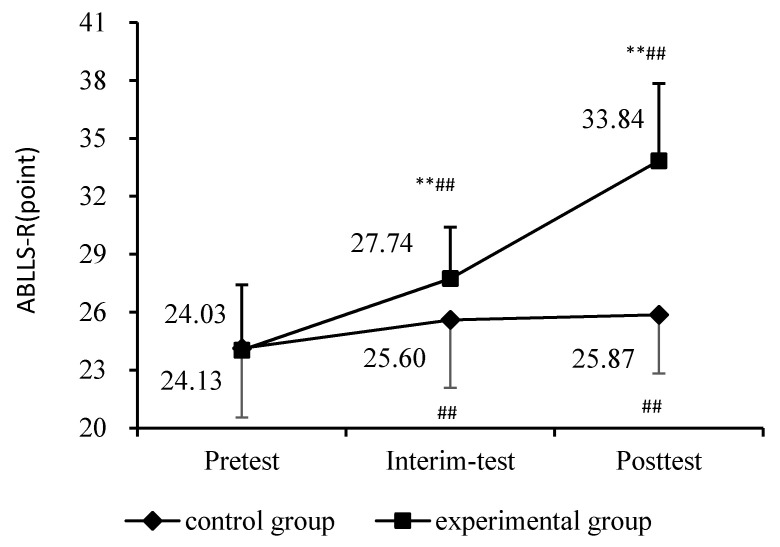
Impact of THR program on the ABLLS-R in the two groups. Compared with the control group, ** *p* < 0.01. Compared with pre-test, ^##^ *p* < 0.01.

As shown in Table 7, no significant difference was found between groups on the SSIS before the intervention. In the interim-test, there was significant improvement for the experimental group compared with the control group (*p* < 0.05). After the THR intervention, a significant increase in social interaction scores was identified in the experimental group (*p* < 0.01). Participants in the THR group had significant improvements in their overall social interaction score from interim-test to post-test. Figure 3 illustrates the trend in social interaction scores for the two groups: it shows that the THR group had a noticeable improvement on the ABLLS-R compared to the control group, which indicated a positive influence of the intervention on the social interaction skills of children with autism.

### 3.2. The Effects of the Therapeutic Horseback Riding Program on Communication

Communication was measured by seven items: says please, responds well to others, speaks in appropriate voice, take turns in conversations, says thank you, makes eye contact when talking, and uses gestures or body language appropriately with others. Repeated ANOVA and dependent samples *t*-tests were conducted to measure the difference among the participants on the seven items measuring communication in the SSIS. As reported in Table 8, there was a significant effect for “says thank you”, “says please” and “makes eye contact when talking” for the experimental group (*p* < 0.05) after the 16 week intervention. In addition, participants in the THR group had significant improvements on six of the seven items, including “says please”, “responds well when others start a conversation or activity”, “speaks in appropriate tone of voice”, “says thank you”, “makes eye contact when talking”, and “uses gestures or body language appropriately with others” over time, from pre-test to post-test.

## 4. Discussion

The current study investigated the effects of a 16-week therapeutic horseback riding program on social interaction among children with autism. The results confirmed the potential role of therapeutic horseback riding as an effective complementary intervention approach for children with ASD. The results of the study suggested that the THR program may be beneficial for children with ASD in the areas of social skills, communication, responsibility, and self-control. This study addresses some limitations of previous studies, with its relatively larger sample size and quasi-experimental design. To achieve better objectivity, this study avoided using parents as the only evaluator: instructors with blinded observers were also used to decrease the potential bias and achieve more reliable results.

Animal-assisted interventions (AAIs) are known to be beneficial for children with developmental disorders, especially children with ASD. AAIs may increase sympathy and understanding of other’s minds through specially structured social interactions. In AAIs, horses are currently recognized as one of the most effective animals for children to work with [5,27]. In an AAI program, a horse can offer a unique outlet for positive social engagement. The results of the SSIS-RS and ABLLS-R in the present study show that the THR program leads to significant improvements in the areas of social interaction and communication. The findings of this study are consistent with previous studies findings that horseback riding has positive influences on social functions [5,14,28,29].

The rhythmic movements of riding horses can stimulate the vestibular system, which can promote the production of speech sounds [15], and a horse moving in a fixed rhythm may play a role in promoting calmness and body coordination. Horses listen to their riders for direction and respond to their rider’s subtle movements and cues during riding. Children need to consciously control their own body movements and behavior on the horse and learn to adjust their body and postures to different positions (upright, prone, supine, forward, backward, and side-bending) during riding. Therefore, the significant improvement in emotion control from the THR group leads us to believe that horses may help children with autism improve their patience and communication, and allow them understand how their behaviors can lead to a good relationship with the horse in a cause-and-effect way through constant practice.

Evidence from the present study indicated that significant improvements in the areas of communication, responsibility, and self-control were observed for the THR group. During the THR intervention, the reactions from the horses or instructors may have stimulated the child’s perception of the surrounding environment. During riding, the horses responded to children’s commands constantly, which can produce non-verbal communications. These child-horse interactions and social practices helped them to better understand others, which is an essential prerequisite for social behavior and communication skills. In addition, during riding, the children were required to maintain postural control and balance, enhancing their attention and self-control. The warmth of the horse’s body and the horse’s rhythmic movements during riding can contribute to calmness, which may reduce irritability and hyperactivity. These psychological mechanisms and physiological processes may account for the characteristics of an AAI horse intervention.

Another possible explanation for the effectiveness of the intervention for children with ASD is the nature of the THR program. The program is structured, goal-directed, progressive, and involves interrelated interactions. The effects of the THR program are achieved through a series of training steps, and an accumulated variety of stimuli. Children are constantly guided to follow directions and give commands through verbal and non-verbal communication (e.g., body language) to their horses. All of the processes require children with ASD to maintain active engagement. During the THR program, every session involves building the horse-child and child-therapist relationship to better facilitate interactions among them. With its fun and enjoyable atmosphere, the THR program provides a type of “Green Environment and Mechanism”, connecting children with horses and the natural environment, and offering a unique type of intervention in which the interplay among the horses, children, and the riding instructors promotes in-depth interactions that address the core impairments of the participants through leisure activities. Since the program is associated with joy, riding horses, and being in contact with nature, it encompasses many specific rehabilitation activities and exercises to meet each participant’s riding goals, and objectives related to the children’s behavior problems. We believe that making the THR program enjoyable ensures it is successful.

In addition, during the intervention program, the participants were accompanied by the same therapist and volunteer throughout. The individualized therapeutic approach of the THR may be more beneficial to children with social and communication deficits. The program is comprised of riding skills, such as mounting, dismounting, halting, steering, turning, and trotting, which focus on promoting participants’ motor, social communication, emotional, and cognitive skills. Following the directions of the ride instructors and volunteers (side walkers) provides the riders with verbal modeling and/or physical prompts as needed, to assist them in the acquisition of riding skills.

All the exercises and activities in the THR program are conducted within a highly structured context, in which social and communication skills are trained efficiently.

In summary, the structured routines, visual cards, scripts, prompts, and natural environment of the THR are considered essential and evidence-based key points for training children with ASD and make it possible to develop and improve their social and communication skills. The findings of this study are not only in line with the results of the existing literature, but also provide evidence of the efficacy of horseback riding programs as a therapeutic approach for children with ASD.

## 5. Conclusions

This study was designed to examine the effects of a 16-week therapeutic horseback riding program on social interaction and communication skills in children with ASD. The findings of this study confirmed the hypothesis that participants in the THR group would demonstrate a significant improvement in social interaction and communication skills compared to the participants in the control group.

These results indicate that the THR program has a positive influence on overall social skill scores on the SSIS. A significant improvement on ABLLS-R scores was also observed in the THR group compared to the control group. A notable improvement on overall social interaction score was found from the interim-test point to post-test. In addition, participants in the THR group showed significant improvements on six out of seven items in their communication evaluations. In summary, after 16 weeks of intervention, the THR program significantly enhanced the sub-domains of social and communication skills in the areas of social interaction, communication, responsibility, and self-control compared to the control group. We agree the comment provided by one of reviewers that AAI approach may be more useful for sensory alternations than communication in children with ASD, however, we didn’t collect data in relation to sensory functional skills in the current study due to the time and other limitations. We will consider studying this area in our future studies.

## Figures and Tables

**Table 1:** The demographic data of the participants.

Variables	Experimental Group	Control Group	Total	$F/t/\chi^{2}$	*p*-Value
Participants	31	30	61	F = 0.144	0.706
Age, y, mean (SD)	7.06(1.50)	7.13(1.36)	7.10(1.42)	t = −0.187	0.852
Gender, n, M/F	21/10	23/7	44/17	$\chi^{2}$=0.604	0.437

**Table 2:** THR intervention program protocol.

Part	Time (min)	Content	Objective
1. Warm-up activities	15	Participants get the visual tools (e.g., pictures, cards) themselves; stretching exercises, arm circles, trunk twists, lunge stretching; put on riding helmets and boots.	Obtain knowledge of horse and riding; social interaction and communication skills
2. Riding and horsemanship skill instruction	20	Learn riding skills (e.g., hand walking the horse, mounting, dismounting, halting, steering, turning, and trotting) and horsemanship skills (e.g., lead and care for the horse); follow the riding instructors and try to practice and interact with the horse; build the participant-horse relationship with the assistance of the riding instructors and volunteers.	Riding skills; motor and balance ability; social skills
3. THR exercises and activities	15	Engage in group exercises and activities on the horse (e.g., red light/green light, cup games, ball/cone games, letter games); reach to touch the horse’s ears or tail during riding.	Social interaction and communication skills
4. Cool-down and reward activities	10	Groom the horses; feed and communicate with the horses (e.g., saying “thanks” and “goodbye”); put away equipment; high five/hugs to riding instructors, volunteers and parents; sing goodbye songs; get rewards (e.g., toys, snacks, stickers).	Rewards; feedback; social interaction and communication skills

**Table 3:** Multivariate tests.

Effect	F	Hypothesis df	Error df	Sig.	ES
Time	38.847	2.000	58.000	0.000	0.573
Time × group	21.057	2.000	58.000	0.000	0.421

**Table 4:** Comparison of social skills across three intervals between groups.

Control Group			Experimental Group			Interaction		
Pretest	Interim-Test	Post-Test	Pre-Test	Interim-Test	Post-Test	F(2,58)	Sig.	ES
44.27 ± 4.31	45.13 ± 4.67	45.43 ± 5.08	44.68 ± 7.48	48.26 ± 6.51 *^##^	50.87 ± 6.47 **^##^	21.057	0.000	0.421

Note. Compared with control group: * *p* < 0.05, ** *p* < 0.01. Compared with Pre-test, ^##^ *p* < 0.01.

**Table 5:** Sub-domains of social skills of the SSIS across time for the THR group and control group.

Index	Control Group (n = 30)			Experimental Group (n = 31)			Interaction		
	Pre	Interim	Post	Pre	Interim	Post	F(2,58)	Sig.	ES
Communication	7.03 ± 1.54	7.17 ± 1.53	7.27 ± 1.46	6.71 ± 1.77	7.74 ± 1.55 ^##^	8.48 ± 1.86 **^##^	10.764	0.000	0.271
Cooperation	7.50 ± 1.41	7.57 ± 1.30	7.63 ± 1.22	7.55 ± 1.61	7.97 ± 1.66 ^#^	8.16 ± 1.73 ^##^	1.532	0.225	0.050
Assertion	4.63 ± 1.10	4.80 ± 1.19	5.07 ± 1.39 ^#^	4.90 ± 1.58	5.23 ± 1.52	5.71 ± 1.47 ^##^	1.212	0.305	0.040
Responsibility	5.87 ± 1.01	6.33 ± 1.21 ^#^	6.13 ± 1.17	6.23 ± 1.23	6.74 ± 1.21 ^##^	7.00 ± 1.24 **^##^	4.168	0.020	0.126
Empathy	5.70 ± 1.02	5.60 ± 1.10	5.53 ± 1.17	5.42 ± 1.29	5.68 ± 1.19	5.90 ± 1.27 ^##^	3.399	0.040	0.105
Engagement	6.47 ± 1.14	6.90 ± 1.09 ^#^	7.03 ± 1.19 ^##^	6.65 ± 1.45	7.52 ± 1.36 ^##^	7.68 ± 1.51 ^##^	1.429	0.248	0.047
Self-control	7.07 ± 1.53	6.77 ± 1.55	6.77 ± 1.55	7.23 ± 1.73	7.39 ± 1.75	7.94 ± 1.55 **^##^	8.928	0.000	0.235

Note. Compared with the control group, ** *p* < 0.01. Compared with Pretest, ^#^ *p* < 0.05, ^##^ *p* < 0.01.

**Table 6:** Mauchly’s Test of Sphericity.

Effect	F	Hypothesis df	Error df	Sig.	ES
Time	62.915	2.000	58.000	0.000	0.684
Time × group	31.076	2.000	58.000	0.000	0.517

**Table 7:** Between-group comparison of overall social interaction scores across three intervals.

Control Group			Experimental Group			Interaction		
Pretest	Interim-Test	Posttest	Pretest	Interim-Test	Posttest	F(2,58)	Sig.	ES
24.13 ± 3.59	25.60 ± 3.52 ^##^	25.87 ± 3.05 ^#^	24.03 ± 3.38	27.74 ± 2.66 **^##^	33.84 ± 4.00 **^##^	31.076	0.000	0.517

Note. Compared with control group: ** *p* < 0.01. Compared with Pre-test, ^#^ *p* < 0.05, ^##^ *p* < 0.01.

**Table 8:** Multivariate analysis on the seven items of communication for two groups.

Index	CONTROL GROUP			Experimental Group				Interaction	
	Pre	Interim	Post	Pre	Interim	Post	F(2,58)	Sig.	ES
Says please	0.67 ± 0.48	0.80 ± 0.41	0.83 ± 0.38	0.52 ± 0.51	0.77 ± 0.43 ^##^	0.94 ± 0.44 **^##^	2.114	0.130	0.068
Responds well when others start a conversation	0.70 ± 0.53	0.83 ± 0.38	0.83 ± 0.46	0.84 ± 0.45	1.00 ± 0.37 ^#^	1.06 ± 0.51 ^##^	0.501	0.608	0.017
Speaks in appropriate tone of voice	1.10 ± 0.66	1.10 ± 0.55	1.30 ± 0.47 ^#^	1.26 ± 0.44	1.35 ± 0.49	1.48 ± 0.51 ^##^	0.310	0.735	0.011
Take turns in conversations	0.87 ± 0.57	0.77 ± 0.57	0.80 ± 0.55	0.74 ± 0.58	0.77 ± 0.56	0.81 ± 0.54	0.816	0.447	0.027
Says thank you	1.23 ± 0.43	1.23 ± 0.43	1.27 ± 0.45	1.23 ± 0.43	1.42 ± 0.50 ^#^	1.61 ± 0.56 *^##^	4.076	0.022	0.123
Makes eye contact when talking	1.10 ± 0.31	1.17 ± 0.38	1.20 ± 0.41	1.10 ± 0.40	1.23 ± 0.50 ^#^	1.39 ± 0.56 *^##^	1.942	0.153	0.063
Uses gestures or body appropriately with others	1.03 ± 0.18	1.07 ± 0.25	1.03 ± 0.32	1.03 ± 0.18	1.19 ± 0.40 ^##^	1.19 ± 0.40 ^##^	2.363	0.103	0.075

Note. Compared with the control group, * *p* < 0.05, ** *p* < 0.01. Compared with Pretest, ^#^ *p* < 0.05, ^##^ *p* < 0.01.

## Data Availability

All data relevant to the study are presented in the article. For further inquiries regarding the reuse of data, please contact the corresponding authors (shchen@eduhk.hk and elite_zhangyj@163.com).
